# Contrasting Profiles of Low-Performing Mathematics Students in Public and Private Schools in the Philippines: Insights from Machine Learning

**DOI:** 10.3390/jintelligence10030061

**Published:** 2022-08-30

**Authors:** Allan B. I. Bernardo, Macario O. Cordel, Minie Rose C. Lapinid, Jude Michael M. Teves, Sashmir A. Yap, Unisse C. Chua

**Affiliations:** 1 Department of Psychology, De La Salle University, Manila 1004, Philippines; 2 Dr. Andrew L. Tan Data Science Institute, De La Salle University, Manila 1004, Philippines; 3 Department of Science Education, De La Salle University, Manila 1004, Philippines

**Keywords:** mathematics achievement, machine learning, Philippines, public vs. private schools, school type, socioeconomic differences, PISA

## Abstract

Filipino students performed poorly in the 2018 Programme for International Student Assessment (PISA) mathematics assessment, with more than 50% obtaining scores below the lowest proficiency level. Students from public schools also performed worse compared to their private school counterparts. We used machine learning approaches, specifically binary classification methods, to model the variables that best identified the poor performing students (below Level 1) vs. better performing students (Levels 1 to 6) using the PISA data from a nationally representative sample of 15-year-old Filipino students. We analyzed data from students in private and public schools separately. Several binary classification methods were applied, and the best classification model for both private and public school groups was the Random Forest classifier. The ten variables with the highest impact on the model were identified for the private and public school groups. Five variables were similarly important in the private and public school models. However, there were other distinct variables that relate to students’ motivations, family and school experiences that were important in identifying the poor performing students in each school type. The results are discussed in relation to the social and social cognitive experiences of students that relate to socioeconomic contexts that differ between public and private schools.

## 1. Introduction

Filipino students were among the lowest performing groups of students among all the participating countries in the 2018 Programme for International Student Assessment (PISA). In mathematics, less than 20% of students demonstrated the minimum proficiency level (Level 2), while more than 50% showed very low proficiency (below Level 1). Scoring below the lowest level of proficiency in the PISA, these Filipino students have been clearly left behind in terms of mathematics education; more than half of this age group of Filipino students have inadequate mathematical skill compared to their peers in other parts of the world. The poor performance in mathematics also varied in degree between the students in public and private schools, where the means were 343 and 395, respectively (Department of Education 2019).

The study aims to identify the factors (personal and contextual) that differentiate the lowest-performing students from the other Filipino students in mathematics in public and private schools in the Philippines. Previous studies have shown that public and private schools in the Philippines have very different environments for learning resources (Trinidad 2020) and for supporting student motivation and engagement (Bernardo et al. 2015), and we explore whether different factors identify low-performing students in each type of school. We use a range of machine learning approaches to analyze the Philippines 2018 PISA data from the student questionnaire and the school questionnaire and analyze the data of students from public and private schools separately. Typical education research in the Philippines studies investigate predictors of achievement at one level of analysis, the machine learning approach allows researchers to consider factors at the student level, their family, the instructional experiences in school, and other school characteristics, and thus reveal a more complex set of factors that identify the students that are left behind in mathematics education in the two types of school in the Philippines.

### 1.1. Filipino Students’ Mathematics Proficiency in PISA 2018

Students’ mathematics proficiency in the PISA assessment relates to the students’ capacity to formulate, use, and interpret mathematics in different contexts, including familiar personal experiences and in broader and more abstract contexts of work, society, and science. Students who are assessed to have good mathematics proficiency are able “to reason mathematically and use mathematical concepts, procedures, facts and tools to describe, explain and predict phenomena” (OECD 2019a, p. 104). The test items were given in combinations of the different mathematical processes, mathematical content, and contexts. The mathematical processes included formulating situations mathematically, employing mathematical concepts, facts, procedures and reasoning, and interpreting, applying, and evaluating mathematical outcomes. Underlying these mathematical processes were fundamental mathematical capabilities such as understanding a problem situation, its tasks, and questions; being able to present, explain and justify a solution; translating and representing the problem and its quantities into a mathematical form; and utilizing mathematical content knowledge and tools to solve the problem and to communicate results (OECD 2019a). 

Six proficiency levels were described to represent the range of mathematics skills, knowledge, and understanding in the 2018 PISA mathematics assessment; the same six levels have been used since mathematics became a focal area of assessment in 2002 (OECD 2019a). Level 2 is considered as the minimum proficiency standard, and less than 20% of Filipino students attained Level 2 proficiency or better. This means that an overwhelming majority of Filipino students score below standard; more specifically, 27% scored at Level 1 proficiency and 54% scored below Level 1 (OECD 2019a). According to the PISA mathematics proficiency guide, Level 1 means: “… students can answer questions involving familiar contexts where all relevant information is present and the questions are clearly defined. They are able to identify information and to carry out routine procedures according to direct instructions in explicit situations. They can perform actions that are almost always obvious and follow immediately from the given stimuli” (OECD 2019a, p. 105). So less than 3 of every 10 15-year-old Filipino students can do math only at that level, and more than half of these students cannot even do those actions. 

While the results suggest that most Filipino high school students are not learning what they are supposed to in mathematics, the situation seems to be worse for the students in Philippine high schools. On average, private school students’ scores were at Level 1 proficiency, while those from public schools were below Level 1. While about 3 of every 10 private school students scored below Level 1 proficiency in mathematics, 6 out of every 10 public school students scored below Level 1.

In a sense, the results are not surprising as the Philippines had been consistently performing poorly in mathematics in the global assessments. It had not been able to improve from the bottom 5 ranks since it joined Trends in Mathematics and Science Study (TIMSS) in 1999 (Mullis et al. 2004). However, the Philippine government chose to participate in PISA 2018 with the aim of gaining knowledge from the international large-scale assessment to help improve the current educational system (National Economic Development Authority 2020). Indeed, the PISA provides data on a wide range of variables that can be studied as possible predictors of successful (or unsuccessful) learning in the different domains. These variables might be interacting in ways that predict either poor or good mathematics achievement. In the next section, we consider the possible types of variables known to be associated with students’ mathematics learning.

### 1.2. Predictors of Mathematics Learning and Achievement

Research has revealed many important predictors of mathematics learning and achievement, and most of the predictors can be classified under one of five broad categories: student factors, family factors, teacher factors, classroom and school factors, and policy factors (Maamin et al. 2021). We will not attempt a comprehensive review of such factors but refer to some that were measured in the PISA 2018 questionnaire and that were included in the analysis for the current study; these factors fall under the first four broad categories, as no policy related factors were included in the student questionnaire of PISA.

Beyond the typical student factors such as gender, cognitive abilities, and metacognitive (Desoete and De Craene 2019; Lindberg et al. 2010), research has confirmed the importance of a range of non-cognitive social psychological factors predictors of student academic success (Lindberg et al. 2010; Kim and Choi 2021). In mathematics achievement, these factors include motivation (Levpušček et al. 2013; Saw and Chang 2018), goal orientations (Dela Rosa and Bernardo 2013), attitudes (Gjicali and Lipnevich 2021), self-beliefs (Damrongpanit 2019; Szumski and Karwowski 2019) and academic emotions (Villavicencio and Bernardo 2013, 2016). There are more specific student factors that relate to these social psychological factors such as the students’ educational and career aspirations; students who have higher career aspirations that also require higher educational qualifications showing stronger motivations related to achieving in mathematics (Watt et al. 2019; Webster and Fisher 2000). On the other hand, poor motivation in learning is associated with students’ absenteeism and tardiness, which are also associated with lower mathematics achievement (Vesić et al. 2021; Gottfried and Ansari 2022).

Family factors that relate to students’ mathematics achievement include factors related to the family’s socioeconomic background, which relates to parents’ education and occupation, as well as the educational resources available in the home (Lam and Zhou 2021; Lombardi and Dearing 2021; Marks and Pokropek 2019). The types of parental support for the students’ learning are also important predictors of students’ achievement (Bernardo et al. 2015; Soenens et al. 2007); parental support also relates to the quality of parent–child relationship (Christenson and Havsy 2004), parental involvement in their children’s learning in mathematics (Hyde et al. 2006; Jay et al. 2018) and expectations of their children’s achievement (West et al. 1998). 

Teachers’ expectations of students also play an important role in students’ achievement in mathematics (Szumski and Karwowski 2019), as do other social and interpersonal teacher factors. Teacher characteristics relate to instructional quality, and both predict higher student achievement in mathematics (Toropova et al. 2019; Wayne and Youngs 2003; Wedel 2021). Teacher characteristics such as teacher preparation (Boyd et al. 2009; Fung et al. 2017), continuing professional development (Desimone 2013; Harris and Sass 2011), mathematical knowledge (Baumert et al. 2010), and teachers’ self-efficacy (Fung et al. 2017; Zee et al. 2018) are some of the qualities that relate to their instructional performance. 

Some teacher factors are also shaped by school-level factors such as school policies on class sizes (Woessmann and West 2006) and support for teachers’ continuing professional development (Desimone 2013). However, other important aspects of the school environment also play an important role in predicting student achievement in mathematics.

The school environment can influence teachers’ and students’ behavior in the teaching and learning process and eventually students’ achievement (Trinidad 2020). A school culture that promotes shared values and norms for learning, high academic standards (Jesse et al. 2004), strong personal bonds between teachers and students showing genuine concern to students for academic success (Mateos et al. 2021) are said to be important predictors of student achievement. Other important predictors include orderly and highly structured schools, classes where rules and procedures are consistently and reasonably enforced (Ilg and Massucci 2003; Pressley et al. 2004), and school environment that encourages student participation in after-class activities (Wigfield et al. 2006). 

However, perhaps one of the most important school factors that predict student achievement relates to the schools’ learning resources (Levpušček et al. 2013; Caponera and Losito 2016). In the Philippines, for example, material constraints and lower teacher resources are associated with lower student attention, lower student respect, more concerns with attendance, bullying, other problematic student behaviors, and student achievement (Trinidad 2020). These resource constraints distinguish the school environments in public and private schools in the Philippines (Lockheed and Jimenez 1994) and also other countries (OECD 2019b), and more importantly, they are associated with achievement gaps (Braun et al. 2006; Carbonaro and Covay 2010). Interestingly, one study showed that school type differences were more pronounced in mathematics achievement compared to other subjects (Lubienski and Lubienski 2006). Other studies found that the achievement gap between private and public schools in the Philippines is also associated with different levels of student motivations and perceived support from parents and teachers (Bernardo et al. 2015) and the higher student selectivity in private schools (Yamauchi 2005). 

### 1.3. The Current Study

The various student, family, teacher, and school factors are also assumed to be interconnected in predicting students’ achievement in mathematics. For example, individual students’ career aspirations are related to their motivational beliefs about math, which are also related to how they perceive their classroom environment (Lazarides et al. 2020). Students’ self-perceptions also interact with the school’s social context in influencing students’ engagement (Wang and Eccles 2013; Fall and Roberts 2012), and their self-beliefs also interact with their socioeconomic status in influencing their mathematics achievement (Bernardo 2021). Thus it is important to try to explore a range of predictors of students’ mathematics achievement to see how they might be working together.

In the current study, we wanted to study the factors that distinguish the Filipino students who perform poorly in the PISA 2018 mathematics assessment from those who met the minimum performance standards. The PISA 2018 obtained self-report data on a wide range of factors –the students, their families, teachers, classes, and schools—that are possible predictors of students’ proficiency in mathematics. Our objective was to identify the models that best identifies the Filipino students who performed poorly in mathematics using machine learning approaches, and we wanted to identify the model for public school students and for private school students. For this purpose, we trained and evaluated different machine learning models on the PH data to determine the best classifier for classifying poor and better performing students. Eighty percent of the data were used to iteratively adjust the model’s parameters during the training phase. Training iterations were terminated based on any of the following conditions: (i) the training performance converges and is less than a preset value, (ii) the validation performance worsens, or (iii) the validation performance does not improve. Each trained model was evaluated using Region of Convergence (RoC and ROC-AUC) scores to determine how well it separates the two categories, standard metrics, e.g., average F1-score to measure its prediction performance, and cross validation to demonstrate its performance on unseen data. By exploring models for identifying poor performing students in mathematics in public and private schools, we hope to identify variables that will point to poor learners’ vulnerabilities that could be the target of interventions. 

## 2. Methods

### 2.1. The Dataset

The data used in the study were derived from the Philippine sample in the PISA 2018 database (publicly accessible at https://www.oecd.org/pisa/data/2018database/ (accessed on 25 November 2021)). The sample was obtained following a two-stage stratified random sampling system. First, 187 schools were randomly selected across the country’s 17 regions, and the students were randomly sampled for each school. The sample comprised 7233 15-year-old students, and of this sample, 18.5% meet the minimum standard defined in the PISA 2018 (i.e., Level 2 or higher) and 26.9% were assessed at Level 1 proficiency. The lowest proficiency group (below Level 1) comprised 54.6% of the sample. 

From the dataset, 96 variables including the estimate for the mathematics achievement (i.e., plausible values 1 or PV1MATH), the school type (SCHLTYPE), and other relevant student-, family-, teacher-, and school-related variables were considered for this exploratory study. We removed three variables with 100% missing values (these were not included in the Philippine version of the survey: ICTSCH, ICTHOME and ST225Q03HA). We also excluded students with more than 50% missing values, decreasing the number of entries to 7091 students. Of this total, 1156 were from private schools (SCHLTYPE = 1 and 2) and 5935 were from public schools (SCHLTYPE = 3).

The remaining variables with missing values in the reduced data set wre imputed using k-nearest neighbor (kNN) algorithm, where k is empirically determined as being equal to 7. PVMATH1 variable was then transformed such that the lowest proficiency students (i.e., students with PVMATH1 < 357.7 or below Level 1), was set to 1 and all the remaining better performing students (i.e., students with PVMATH1 ≥ 357.7 or Levels 1 to 6) is set to 0.

Normalization per variable, except for SCHLTYPE, was then performed such that each variable range is from 0 to 1. We further reduce the number of variables by removing variables with strong positive or negative correlation, i.e., |rho| > 0.75, resulting in a more condensed dataset with 58 variables. More details on the data description can be found in the Supplementary File.

### 2.2. Machine Learning Modeling

Our objective was to discover the key variables that characterize the poor performing students, or more specifically that differentiate them from the better performing Filipino students in mathematics. Machine learning (henceforth, ML) algorithms are typically used to discover the intrinsic and highly complex relationship of the input data and output data. An exhaustive search approach on the hyperparameters of different ML models, namely Logistic Regression, Multilayer Perceptron (MLP), Support Vector Machine (SVM), Decision Tree and Random Forest, was performed to zero in on the most optimal model for the classification task. Table 1 summarizes the hyperparameters considered in the exhaustive search.

**Figure 1:**
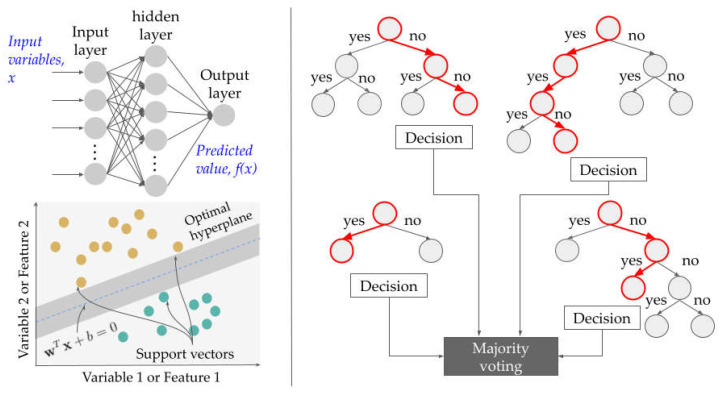
Illustration of a Multilayer perceptron (**top left**), Support Vector Machine with linearly separable data (**bottom left**), and a Random Forest (**right**) with four Decision Tree predictors.

The first two ML models considered, i.e., Logistic Regression and MLP, are the perceptron-type models whose generic representation is shown in Figure 1, top left. Each node *h* of the hidden and output layers, *l*, compute for the activation, $z_{h}^{\left(l\right)}$, given the previous activations $z^{\left(l-1\right)}$, such that

(1) $$z_{h}^{\left(l\right)}=f_{h}^{\left(l\right)}\left(z^{\left(l-1\right)}\right)$$

(2) $$f_{h}^{\left(l\right)}\left(z^{\left(l-1\right)}\right)=a{\left(w^{\left(l\right)T}\;z^{\left(l-1\right)}\right)}_{\;}^{\;\;}$$

(3) $$z^{\left(l-1\right)}=\mathrm{input}\;x\;\mathrm{for}\;l-1\;=\;-1$$

where the superscript indicates the layer with *l* = 0 to *L* and *L* is the number of hidden layers. Note that *L* = 0 for logistic regression. Also, the subscript indicates the node in a particular layer with *h* = 0 to *H* and *H* is the number of nodes for a particular layer. The operator *a* is the activation function and *^T^* is the transpose operation. The parameters that define the model are the weight connection values, **w**. Equations (1) and (2) are computed from input to output using the weight connection values. To adjust these during training, a backward pass, i.e., from output to input is performed as guided by the prediction error every iteration and the learning hyperparameters. 

Another ML model considered was the kernel-based type ML model, particularly SVM, illustrated in Figure 1, bottom left. SVM looks for the most optimal decision plane to optimally separate data into different categories. The SVM decision plane is defined by **w***^T^***x** + *b* = 0, where **x** is the input feature vector and **w** is the weight vector. The training objective is to look for representative data or samples, called the support vectors, that provide maximum margin between the decision boundary and these support vectors. For non-linearly separable data, the variable space is transformed to higher dimension through transformation kernels. The nonlinearity in kernels can be varied using the kernel parameters. See Table 1 for the evaluated hyperparameters.

Finally, the last ML models considered were tree-based models which are more powerful for data whose normality cannot be assumed. Tree-based models, e.g., a Random Forest (see Figure 1 right), split the data from the top down to its decision nodes, grouping the data into the most homogeneous “sub-nodes”, based on their attributes. A Decision Tree is very intuitive and applicable for explaining key variables in the prediction decision. However, these models are prone to overfitting. The Random Forest model addresses this issue by utilizing several Decision Tree estimators. The datasets in Random Forest are bootstrapped and features are randomly sampled per estimator to form its training data. The decisions of these trees are then combined using majority voting. The quality of the overall data split is monitored using Information Gain which measures the impurity reduction.

The classification task was performed for 7091 participants from private schools and public schools. For each group of participants, samples were randomly shuffled and split into 80-20 training sets, respectively. For private schools, there were 1238 training samples after data balancing using oversampling and undersampling (i.e., 619 for each class, 0 and 1) and 232 test samples (72 for class 1 and 160 for class 0). For public schools, 5316 training samples were used after data balancing using oversampling and undersampling (i.e., 2658 each for class 0 and 1), and 1419 testing samples were set aside. Finally, the exhaustive search for the best ML model and the corresponding set of hyperparameters was performed. Each training in the exhaustive search carried out five-fold cross validation, used 600 iterations and reported the average precision, recall, F1-score and accuracy. More details on the data description can be found in the Supplementary File.

## 3. Results

### 3.1. Machine Learning Modeling Results

**Figure 2:**
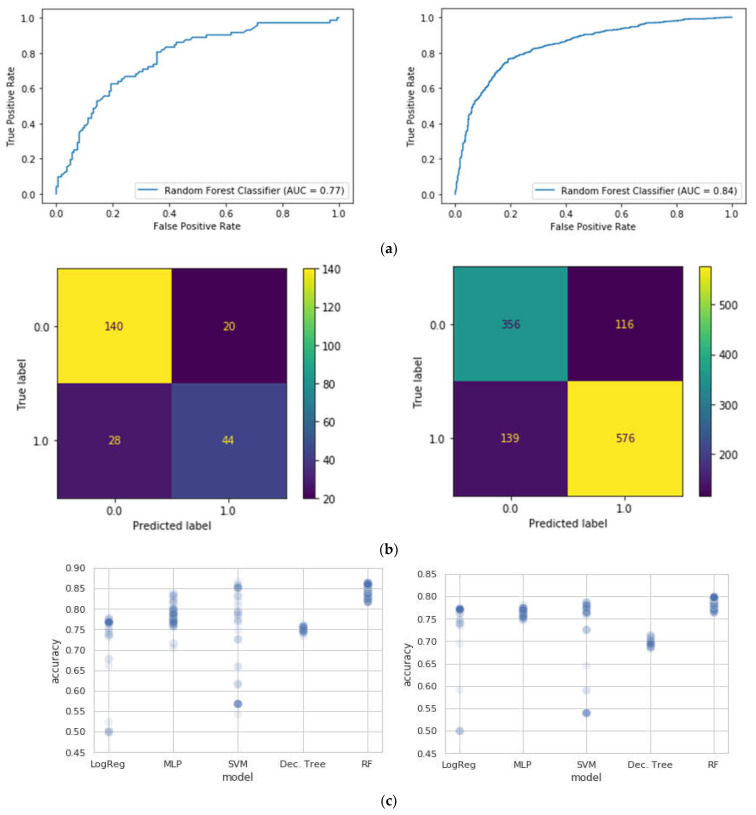
(**a**) Area under the ROC curve (AUC) indicators for the private (**left**) and public (**right**) school participants. AUC score indicates how well separated are the classes 0 and 1 in the Random Forest classifier. (**b**) Confusion matrix for the Random Forest Classifier model for the private (**left**) and public (**right**) school participants. (**c**) a cursory look at the accuracy of the different ML models in the exhaustive search for best hyperparameters for the private (**left**) and public (**right**) school participants. Note that RF performs better than other ML models in terms of performance consistency regardless of the hyperparameters.

The results suggest that the best classifier for the task for both private and public schools is the Random Forest classifier. Table 2 and Figure 2 summarize these results.

### 3.2. Most Important Variables

To identify the level of importance with which poor and better performers in mathematics were classified, we used Shapley Additive Explanations (or SHAP values) which tell how to fairly distribute the prediction outcome among the features (Lundberg and Lee 2017). The SHAP value is the mean marginal contribution of a feature value across all possible feature groups. It produces a ranked list of several features in descending order, indicating the degree of significance of the features.

**Figure 3:**
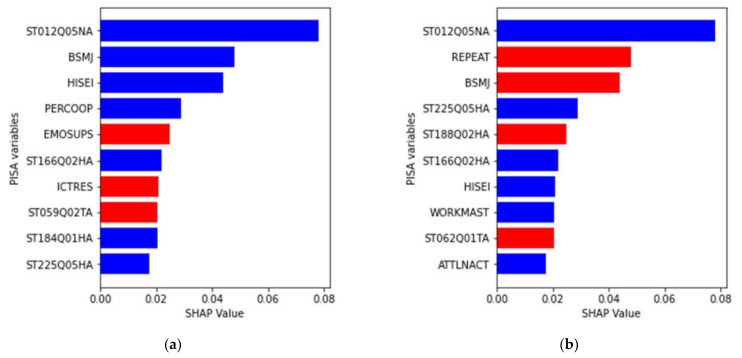
Top 10 most significant variables (in descending order) in the Random Forest model classifier for (**a**) private school participants and (**b**) public school participants. Red bars represent direct relationships with identifying the poor performing students while blue bars represent inverse relationships with identifying poor performing students. SHAP values represent the level of variable importance relative to other variables.

Initial works used top 10 (Chen et al. 2021), top 15 (Dong and Hu 2019; Bernardo et al. 2021a), and top 20 (Chen et al. 2021; Dong and Hu 2019) variables in their feature importance analysis. In this work, to manage complexity in comparing the key variables for private and public student performance classification, the 10 most significant features for the public and private school groups are analyzed and illustrated in Figure 3. 

Four variables were consistent significant features for both models for the private and public school students: ST012Q05NA, ST225Q05HA, ST166Q02HA, and HISEI. All four had inverse relationships with identifying poor performing students, which means that lower scores in the variables were associated with better identification of poor performing students in mathematics.

ST012Q05NA is the questionnaire item that inquired about how many mobile phones that have internet access there are in the student’s home. So having lower values on this item strongly identified poor performing students in both private and public schools; presumably, these are students with no internet access and/or no mobile phones at home. ST225Q05HA is the item that asked the students if they expect to complete a vocational degree after high school. So students in both private and public schools who do not expect to complete this postsecondary credential are more likely to be identified as poor performing in mathematics. ST166Q02HA, is a specific item in a set of that assess students’ view of appropriate responses to receiving a possible SPAM email message. This item refers to checking the email address of the email’s sender. Students who say that checking the email address is not an appropriate response are more likely to be identified as poor performing in mathematics, both for private and public school groups. The final important variable was HISEI or the parents’ occupational status, which were scored using the international socioeconomic index of occupations (Ganzeboom 2010). Students whose parents had lower status occupations were more likely identified as poor performing in mathematics in both public and private schools.

One variable—BSMJ—was important in both private and public school models but in different directions. BSMJ is the variable that asked the students to indicate their expected job when they are 30 years old, and these were also scored using the international socioeconomic index of occupations (Ganzeboom 2010). For the private school group, higher expected occupational status negatively indicated the poor performing students; that is, students who indicated lower expected occupations were more likely to be poor performing studies. However, for the public school, the result was reversed. Higher expected occupational status directly indicated the poor performing students; students who expected higher occupational status were more likely to be poor performing students in mathematics. This is an unexpected but interesting finding that might reflect on how high school students from the two school types think about how their education (and possibly how their mathematics education) relates to the jobs they are likely to have in the future. We discuss this result in more detail in the Discussion section but use this divergent result to begin presenting the other different important variables for private and public school students.

For public school students, three non-cognitive motivation-related variables were important in identifying the poor performing students in mathematics: WORKMAST, ATTLNACT, and ST188Q02HA. WORKMAST and ATTLNACT are both indexes computed based on responses to a set of items, and both inversely related identifying poor performing mathematics students. WORKMAST represents the motivation and persistence to master given learning tasks, whereas ATTLNACT represent the value of schooling, specifically, the importance of trying hard at school to get a good job or into a good college in the future. So students who had low scores in these two motivational variables are more likely to be identified as poor performers. ST188Q02HA is a specific self-efficacy item that states, “I feel proud that I have accomplished things” and students who had a high score on this item were more likely to be identified as poor performing. The result seems odd as feeling proud about one’s accomplishment is not an emotion that one would associate with poor performance, but that is what the results indicate. Two other important variables related to the students’ school record. REPEAT was a categorical variable that indicated whether the student had previously repeated a grade level, while ST062Q01TA referred to how often the student skipped a whole day in school during the past two weeks. Both variables positively identified poor performing students in public schools. 

For private school students, a different set of non-cognitive engagement-related variables were important in identifying the poor performing mathematics students: PERCOOP, EMOSUPS, and ST184Q01HA. PERCOOP represents the students’ perception that cooperation is encouraged in their school and was inversely related to identifying poor performing students; students’ who reported the cooperation is not encouraged in their private school were identified as poor performing in mathematics. EMOSUPS was the index of emotional support from parents, which was directly related to identifying poor performing student; students who report having parents who were emotionally supportive were likely to be identified as low performing. ST184Q01HA is the single-item measure of fixed mindset for intelligence (i.e., the belief that one’s intelligence cannot be changed) and was inversely related to identifying poor performing students. Therefore, students who do not believe that intelligence is fixed are more likely to be identified as poor performing in mathematics. The other two important variables were both directly related to identifying poor performing students. ICTRES was an index of available ICT resources in the students’ home, and this is a broader set of resources compared to ST012Q05NA, which were mobile phones with internet. Interestingly, students with more ICT resources were identified as poor performing, which may indicate that the ICT devices may not necessarily be used to support learning in mathematics. Finally, ST05902TA which referred to the number of required class periods for mathematics per week; fewer required class periods identified poor performing students. 

## 4. Discussion

The aim of the study was to use machine learning approaches to pinpoint important variables that can be used to identify the poor performing Filipino students in public and private schools, with the goal of possibly identifying those factors that make students more vulnerable to poor achievement in mathematics. We analyzed data from students in public and private schools separately, assuming that there might be different identifying factors given the different environments and contexts of the two types of schools in the Philippines. Random Forest classifiers generated the best performing models for both private and public school groups, and Shapley Additive Explanations (SHAP) analysis pointed to notable similarities and differences in the top ten variables that identified poor performing students in each school type. 

For students in both private and public schools, variables that indicate resource constraints identify poor performing students, but the constraint goes beyond material disadvantage and relates to aspirational constraints as well. However, the poor achieving Filipino students are also identified as having lower expectations of completing a postsecondary vocational degree and lower expected occupations when they become adults and have parents who have low status occupations also. Previous studies have noted how the occupational status of parents also tends to be associated with students’ own educational and occupational aspirations (Al-Bahrani et al. 2020; Gutman and Schoon 2018), with parents’ occupational status typically associated with socioeconomic status, as well (Lee and Byun 2019; Ng and Choo 2021). The relationship of lower student educational and occupational aspirations with lower achievement is typically associated with less positive motivations and less engagement (Al-Bahrani et al. 2020; Watt et al. 2019). 

In the case of the public school students, these less positive motivations were among the important variables in the model; poor performing public school students were identified by the low importance they ascribe to trying hard at school get a good future (ATTLNACT) and lower persistence to master given tasks (WORKMAST). In the context of the poor performing students’ low educational and occupational aspirations, it seems to make sense that feeling proud of their school achievement (ST188Q02HA) even if their performance is poor also identifies the poor performing students in public schools. That is, given their limited expectations, they may be quite satisfied by their limited achievement, as well. 

The variables associated with low educational/occupational aspirations and poor performance in mathematics have a different dynamic among the private school students. As mentioned earlier, higher emotional support from parents identified the poor performing private school students. This might reflect a parenting style that provides unconditional emotional support to the children, which has been shown to be an important factor in academic success among disadvantaged students (Osman et al. 2021). Low fixed mindset (or higher growth mindset) is also said to be associated with higher achievement in mathematics (Hwang et al. 2019), but it also identified poor performing mathematics students in private schools. It is possible that unconditional emotional support and the growth mindset are constructed differently by these private school students, in ways that do not relate to being more motivated and engaged to achieve; these possibilities could be investigated in future research. 

The SHAP analysis for both private and public schools show that fewer (or no) mobile phones with access to the internet had a very strong impact on identifying the poor performing student in both private and public schools. This result is consistent with studies showing how mobile phones can promote more positive motivations and higher achievement in mathematics (Güler et al. 2022; Yoon and Yun 2021). However, among private school students, having more ICT resources at home also identifies the poor performing students, which may be explained by how such higher access to ICT is used for non-educational purposes. There are some studies that do show how use of ICT for leisure is negatively associated with mathematics achievement (Petko et al. 2017; Skryabin et al. 2015). So while, lack of access to a specific form of ICT resources seems to adversely affect students, in the case of private students, having more ICT resources seems to also have adverse effects on achievement, or at least seems to identify some of the poor performing students in mathematics.

Among public school students, skipping class days was also associated with identifying poor performance in mathematics. A qualitative study of absenteeism in the Philippines found varied reasons why students skip their classes, including feeling helpless in their classes, having mixed priorities, and unappealing learning environments (Clores 2009), but among students from lower income families, one reason for skipping classes is not having money for transportation to go to school, or they may be too hungry to go to school on an empty stomach (Jabar 2021). In the case of public school students, these different reasons might be intersection, and having an adverse impact on the students’ learning of mathematics.

We also note that there is one variable related to the students’ metacognitive abilities related to potential misinformation on emails. This result that was found in both private and public school students suggests that there might be some specific metacognitive skills that are lacking or not well developed among the Filipino students who are poor in mathematics (Desoete and De Craene 2019). 

Note that other than the last mentioned variable, most of the important variables that identify the poor-performing students in mathematics are variables that relate to the resource limitations (i.e., associated with more disadvantages socioeconomic conditions) and associated motivations and aspirations. Indeed, the poor performing students tend to have parents with low status occupations, who do not have mobile phones that have internet access and may not have as sharp metacognitive skills in dealing with possible false information online, and who have lower educational and occupational aspirations for themselves. In the case of the students from public schools, the relative deprivations seem to be also associated with skipping classes, weaker motivations to persist in task mastery, lower appreciation of the value of education to succeed in the future, and engagement. 

In the case of private school students, there are identifying variables that do not relate to resource limitations, and are instead, known to be associated with higher achievement in the research literature (e.g., ICT resources at home, emotional support from parents, growth mindset, and classrooms that encourage cooperation). Thus, among private school students, there might be students who perform poorly in mathematics for other reasons. That is, these students are doing badly in mathematics even as there are aspects of their learning experiences and environments that, in theory, should be helping them do well. Earlier we suggested that these students might be giving different meanings to these positive aspects. For example, the emotional support from their parents might be constructed as being unconditional regardless of how well they do in school and a signal not to try to work harder to achieve. Their low fixed mindset about intelligence might suggest that they do not see their poor performance in mathematics as defining their intelligence and sense of worth. Furthermore, their perception that the school encourages cooperation among learners might be recognized as an opportunity to rely on others to get by in their mathematics courses. These interpretations are speculative and will need to be further probed in future research studies.

However, what the above discussion points to is that while there are common variables that identify poor performing students across schools, there are specificities in the experience and context of private school students that suggest different identifying variables and vulnerabilities. Indeed, it is possible that if we look at a longer list of important variables, we might also find such specificities in the vulnerabilities in different public school contexts, as well. 

We applied machine learning techniques to try to identify the variables that identify the poor performing Filipino students in mathematics, with the assumption that such variables will point to possible vulnerabilities or risk factors associated with poor learning in mathematics. The foregoing discussion points to most factors that relate to the student, but there are also important predictors that relate to the student’s family, and the results also indicate that school type (i.e., public or private) is a factor that also comes into the picture. However, machine learning approaches cannot actually reveal how variables at the student level interact with the variables at the family and school levels. Indeed, machine learning approaches can find the most accurate predictive models but treat variables equally without any levels. As such machine learning approaches cannot be used to test explanatory models that specify theoretical propositions regarding relationships among variables at different levels (Shmueli 2010). To test such explanatory models, statistical analysis to test multilevel models that propose interactions among factors at different levels and also control variables at different levels. For PISA mathematics data, other researchers have used hierarchical linear modeling techniques (Hu et al. 2018; Osborne and Ma 2020) and multilevel structural equation modeling (Bernardo et al. 2021b) to study such multilevel models. Given the limitations of machine learning in this regard, other have suggested combining machine learning and statistical approaches to capitalize on the strengths of both approaches for studying large scale assessment data like the PISA (Lezhnina and Kismihók 2022).

## 5. Conclusions

Our study points to a cluster of resource constraint-related variables that include motivational and social cognitive elements, and also possible distinct convergences of factors for those in public and in private schools. We note that in the Philippines’ Department of Education’s report on the 2018 PISA results (Department of Education 2019), the country’s educational policy decision makers reiterated their four focal thrusts in the efforts to improve student learning: curriculum review and update, improving the learning environment, teacher “upskilling and reskilling,” and engaging stakeholders for support. These thrusts have been the focal points of improving mathematics education in the Philippines for many years now (Ogena et al. 2018). Our results call attention to the need to go beyond curricular and instructional factors, as there are elements of the students’ social and psychological experiences in school that are important identifiers of poor performing students. While improving the learning environment might be a good entry point to begin addressing these vulnerabilities, the specific ways of enhancing these learning environments might require a deeper understanding of the particular social and psychological factors that make school environments less effective.

## Figures and Tables

**Table 1:** List of the considered ML models and the different hyperparameters during the grid search. Hyperparameters define the complexity of the ML and each model’s learning performance during the training.

ML Models	Hyperparameters
Logistic Regression	solver: newton-cg, lbfgs, liblinear penalty: none, l1, l2, elasticnet c: 1 × 10^−5^, 1 × 10^−4^, 1 × 10^−3^, 1 × 10^−2^, 1 × 10^−1^, 1, 10, 100
MLP	hidden layer sizes: (10, 30, 10), (10, 30), (32, 32), (10, 10, 10, 10) activation: tanh, relu, logistics solver: stochastic gradient descent, adam alpha: 1 × 10^−4^, 5 × 10^−3^, 5 × 10^−2^ learning rate: constant, adaptive
SVM	kernel: radial basis function, polynomial gamma: 1, 1 × 10^−1^, 1 × 10^−2^, 1 × 10^−3^, 1 × 10^−4^ *c*: 1 × 10^−1^, 1, 10, 100, 1000
Decision Tree	criterion: gini, entropy max depth: 4, 5, 6, 7, 8, 9, 10, 11, 12, 15, 20, 30, 40, 50, 70, 90, 120, 150
Random Forest	criterion: gini, entropy number of estimators: 200, 500 max features: auto, sqrt, log2 max depth: 4, 5, 6, 7, 8, 9, 10, 11, 12, 15, 20, 30, 40, 50, 70, 90, 120, 150

**Table 2:** Summary of best validation performance per ML model after grid search. Text in bold indicates best ML model performance for a specific metric and school type. For both participants from private and public schools, the best classifier is the Random Forest in terms of accuracy.

School Type	ML Model	Validation Performance				Hyperparameters Optimal Values
		Precision	Recall	F1-Score	Acc	
Private	Logistic regression	0.63	**0.75**	**0.68**	0.74	C: 1; penalty: l2; solver: newton-cg
	MLP	0.67	0.56	0.61	0.73	activation: ‘relu’; alpha: 0.005, hidden_layer_sizes: (32, 32) learning_rate: ‘constant’, solver: ‘adam’
	SVM	0.67	0.02	0.04	0.63	C: 10; gamma: 1; kernel: rbf
	Decision tree	0.54	0.54	0.54	0.72	criterion: gini; max_depth: 12
	Random forest	**0.69**	0.61	0.65	**0.79**	criterion: ‘gini’; max_depth: 20 max_features: log2 n_estimators: 500
Public	Logistic regression	0.81	0.75	0.78	0.75	C: 1; penalty: l1; solver: liblinear
	MLP	0.80	0.75	0.78	0.74	activation: ‘relu’; alpha: 0.05; hidden_layer_sizes: (32, 32) learning_rate: ‘constant’, solver: ‘sgd’
	SVM	0.75	0.76	0.75	0.70	C: 100; gamma: 0.1; kernel: rbf
	Decision tree	0.76	0.76	0.76	0.71	criterion: gini; max_depth: 6
	Random forest	**0.81**	**0.78**	**0.79**	**0.79**	criterion: ‘gini’; max_depth: 15 max_features: auto n_estimators: 200

## Data Availability

The data analyzed in this study are available in the PISA 2018 Database page on the website of the Organisation for Economic Co-operation and Development at https://www.oecd.org/pisa/data/2018database/ (accessed on 25 November 2021).

## Supplementary Materials

The following supporting information can be downloaded at: https://www.mdpi.com/article/10.3390/jintelligence10030061/s1, File: Additional Data Description.
