# Supplementary material for: Contrasting Profiles of Low-Performing Mathematics Students in Public and Private Schools in the Philippines: Insights from Machine Learning
Source: J Intell. 2022 Aug 30;10(3):61. doi: 10.3390/jintelligence10030061 (PMC9504801; doi:10.3390/jintelligence10030061)
Supplement: Supplementary file 1 [file jintelligence-10-00061-s001.zip › jintelligence-1817350-supplementary.pdf]

Contrasting Profiles of Low-Performing Mathematics Students in Public and Private Schools in the Philippines: Insights from Machine Learning

Supplementary Materials

Additional Data Description

The PISA dataset measuring math proficiency of 7233 Filipino students is used for this study. As summarized in Figure 1-(a), around 54.60% of the students belong to level 1 math competency, roughly 26.92% have level 2 math competency, while approximately, only 18.50% of the students have higher than level 2 math competency. The top variables with the highest number of missing variables are shown in Figure 1-(b).

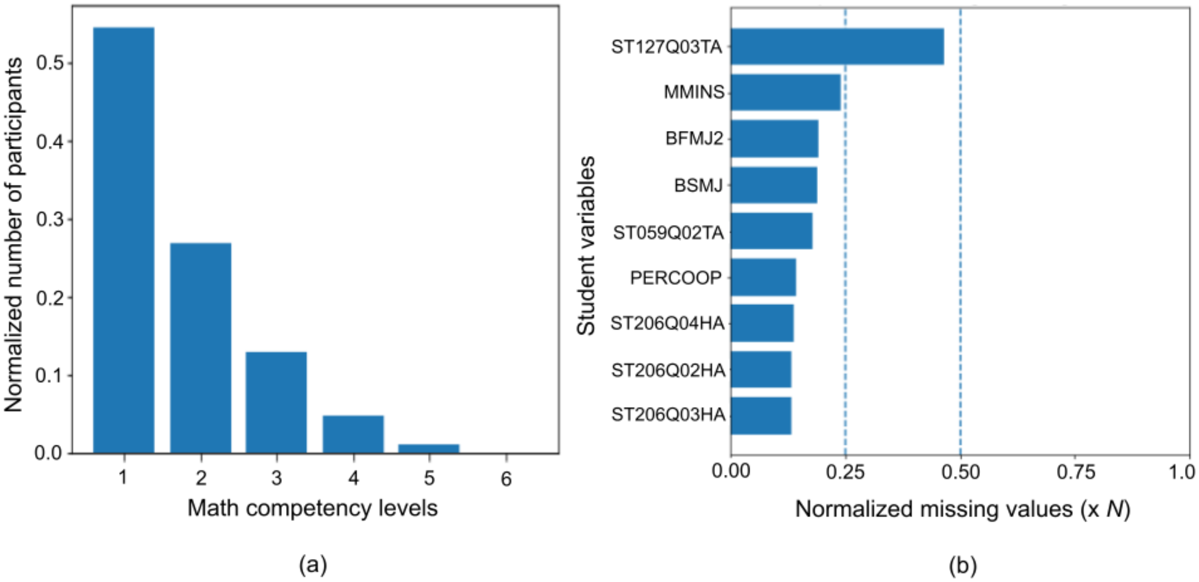

Figure S1. (**a**) Normalized distribution of math competency levels of FIlipino students. Around 54.60% of students have level 1 math competency while 45.50% have level 2 and higher math competency. (**b**) Variables with missing values. Note that these variables have missing values for less than 50% of the student participants.

We divided the Philippine data into two groups, i.e. data from private schools (SCHTYPE = 1 or 2) and data from public schools (SCHTYPE = 3). The number of students from each school type, and the distribution of students with poor and good performance are summarized in Table 1. Each dataset was further split into training and test sets. The training data were used for training the machine learning models while the test data were used for evaluation. To minimize the possibility of having high variance and high bias models, we performed data balancing by oversampling using SMOTE and undersampling using Tomek Links method. The final number of training data after balancing are detailed in Table 2.

Table S1. Data distribution of train and test sets with 80%-30% split. The total number of processed data is 7091. Note the imbalance in the number of training samples for the good and poor performing students.

School type	Data split	Good performance (Level ≥ 2)	Poor performance (Level = 1)	TOTAL
Private school	Training data	636	288	924
	Test data	147	85	232
	**TOTAL**	**783**	**373**	**1156**
Public school	Training data	1989	2759	4748
	Test data	489	698	1187
	**TOTAL**	**2478**	**3457**	**5935**

Table S2. Training data distribution after balancing using the SMOTE-Tomek Links algorithm. SMOTE and Tomek Links are oversampling and undersampling methods respectively.

	Good performance (Level ≥ 2)	Poor performance (Level = 1)	TOTAL
Private school	619	619	1238
Public school	2658	2658	5316
